# Interaction-driven breakdown of Aharonov–Bohm caging in flat-band Rydberg lattices

**DOI:** 10.1038/s41567-024-02714-7

**Published:** 2025-01-10

**Authors:** Tao Chen, Chenxi Huang, Ivan Velkovsky, Tomoki Ozawa, Hannah Price, Jacob P. Covey, Bryce Gadway

**Affiliations:** 1 Department of Physics, University of Illinois at Urbana-Champaign, Urbana, IL USA; 2 Advanced Institute for Materials Research (WPI-AIMR), Tohoku University, Sendai, Japan; 3 School of Physics and Astronomy, University of Birmingham, Birmingham, UK; 4 Department of Physics, The Pennsylvania State University, University Park, PA USA

**Keywords:** Quantum simulation, Condensed-matter physics, Exotic atoms and molecules

## Abstract

Flat bands in condensed matter systems can host emergent states of matter, from insulating states in twisted bilayer graphene to fractionalized excitations in frustrated magnets and quantum Hall materials. A key phenomenon in certain flat-band systems is Aharonov–Bohm caging, where particles become localized due to destructive interference caused by gauge fields. Here we report on the experimental realization of highly tunable flat-band models populated by strongly interacting Rydberg atoms. By employing synthetic dimensions, we engineer a flat-band rhombic lattice with twisted boundaries and explore the control of Aharonov–Bohm caging during non-equilibrium dynamics through a tunable gauge field. Microscopic measurements of Rydberg pairs reveal the interaction-driven breakdown of Aharonov–Bohm caging in the limit of strong dipolar interactions, where lattice bands mix. In the limit of weak interactions, where caging persists, we observe effective magnetism arising from the interaction-driven mixing of degenerate flat-band states. These observations offer insights into emergent phenomena in synthetic quantum materials and expand our understanding of quantum many-body physics in engineered lattice systems.

## Abstract (web summary)

The effect of strong interactions on the physics hosted by flat bands remains largely unexplored in atomic systems. An experiment in a synthetic flat-band lattice now demonstrates an interaction-driven transition from localization to delocalization.

## Main

Frustration, resulting in degenerate eigenstates and flat energy bands that are sensitive to perturbations, underlies many of the phases and phenomena that define the forefront topics of condensed matter physics. This includes the fractionalized quasiparticles of quantum Hall matter^1^ and spin models^2^, the intertwined orders of heavy fermion compounds^3^, and the rapidly growing field of moiré materials^4^. Flat-band lattices^5^, in which the frustration of electronic wavefunctions or interacting spins leads to perfectly degenerate energy bands, have played a specifically important role in enriching the understanding of itinerant ferromagnetism^6,7^ and lattice analogues^8,9^ of fractional Hall states^1,10^.

Recently, researchers have used the tools of synthetic quantum matter to engineer frustration^5,11^ in electronic^12^, photonic^13^ and atomic^14–19^ systems. The tunability of such platforms has even enabled the realization of Aharonov–Bohm (AB) caging^18,20–23^, a condition in which all bands become perfectly flat in the presence of a gauge field due to destructively interfering tunnelling pathways. Under AB caging, delocalized Bloch waves are transformed into compact localized states (CLSs).

Although many exciting questions relate to how interactions can lead to emergent physics in an AB-caged flat-band lattice^24–27^, realizations with light and atoms have been restricted to the non-interacting limit. Recently, experiments with superconducting qubits and Rydberg atoms have begun to probe interaction effects on a single frustrated plaquette^28,29^. Here we explore the breakdown of AB caging in a flat-band lattice due to dipolar Rydberg interactions. We engineered tunable flat-band tight-binding models with microwave-driven Rydberg synthetic lattices^29–33^. For single atoms, we directly observed the AB caging and the independence of the dynamics on the twist phase. For pairs, we observed the predicted^24^ breakdown of AB caging due to interactions, with interaction-enabled delocalization for intermediate interaction strengths and the slow dynamics of bound pairs in the limit of strong interactions. Finally, we characterized the emergent magnetism of flat-band pseudospin states due to dipolar interactions.

## AB caging in rhombic flat-band lattices

We constructed flat-band lattices using the nascent approach of Rydberg synthetic dimensions^29–36^. Here, Rydberg states play the role of lattice sites, and the elements of an effective tight-binding model—the potential energy landscape and (complex) hopping terms—can be finely tuned through spectroscopic control over the transitions between the Rydberg levels^32^. Dipolar interactions^37^ between neighbouring Rydberg atoms in an array further introduce correlated-pair-tunnelling terms along the synthetic dimension^29,38^.

**Fig. 1:**
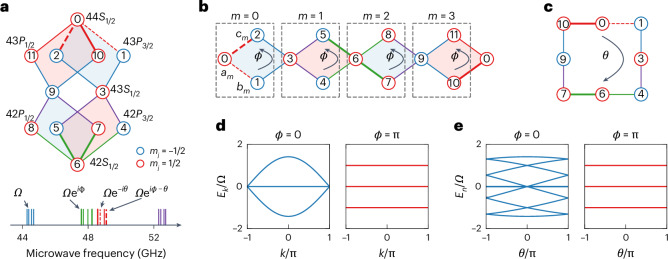
Implementation of a twisted rhombic flat-band lattice in Rydberg synthetic dimensions. **a**, A set of Rydberg states (top) are coupled with engineered multi-tone microwaves (bottom) to form a twisted rhombic structure. The red and blue circles correspond to *m*_*j*_ = 1/2 and −1/2 sublevels in each S or P Rydberg state manifold, respectively. Both the plaquette flux *ϕ* and twist phase *θ* were introduced by tuning the phases of the microwave components driving the transitions (indicated by bold and dashed lines) relative to those driving the other transitions (thin solid lines). The coupling strength for each transition was calibrated by pairwise Rabi dynamics (Methods and Supplementary Information) and set to a common value ~*Ω*/2. **b**,**c**, Expansion (**b**) and side view (**c**) of the twisted structure, depicting unit cells (with index *m*) and sublattice sites labelled as *a*_*m*_, *b*_*m*_ and *c*_*m*_. The plaquette flux was controlled by adding another phase *ϕ* to four transitions ($\left\vert 2\right\rangle \leftrightarrow \left\vert 0\right\rangle$, $\left\vert 10\right\rangle \leftrightarrow \left\vert 0\right\rangle$, $\left\vert 6\right\rangle \leftrightarrow \left\vert 5\right\rangle$ and $\left\vert 6\right\rangle \leftrightarrow \left\vert 7\right\rangle$, bold lines) relative to that of the other transitions in each plaquette. For *ϕ* = 0 and π, the twist phase *θ* could be tuned by introducing another phase −*θ* to the transitions $\left\vert 1\right\rangle \leftrightarrow \left\vert 0\right\rangle$ and $\left\vert 2\right\rangle \leftrightarrow \left\vert 0\right\rangle$. **d**, Eigenenergy bands of the extended rhombic lattice for plaquette flux *ϕ* = 0 (left) and π (right) versus quasimomentum *k*. **e**, Fourfold-folded eigenenergy spectrum of the twisted rhombic lattice for *ϕ* = 0 (left) and π (right) versus the twist phase *θ*. Here *n* is the eigenstate index.

Extending earlier work^29–31^, we used up to 12 Rydberg levels to engineer intricate three-dimensional-like lattice structures with kinetic frustration and twisted^39^, periodic boundary conditions as depicted in Fig. 1a. Using this synthetic lattice, we explored both the single-atom and correlated-pair dynamics by preparing arrays of isolated atoms and isolated atom pairs. We prepared our Rydberg atom samples using the methods detailed in refs. ^29,40^, based on the loading, cooling and imaging of ^39^K atoms in optical tweezer arrays, followed by their excitation to the Rydberg level $n{L}_{J,{m}_{J}}=42{S}_{1/2,1/2}$ (labelled by the synthetic ‘site’ index 6 in Fig. 1a), where *n*, *L*, *J* and *m*_*J*_, respectively, correspond to the principle number, orbital angular momentum, total angular momentum and projection of total angular momentum along quantization axis.

Figure 1a depicts how we created our twisted-boundary rhombic lattice structure by simultaneously driving 16 different microwave transitions between the magnetic sublevels (*m*_*j*_ = ±1/2 states) of the *S*_1/2_, *P*_1/2_ and *P*_3/2_ manifolds for principal quantum numbers *n* ∈ {42, 44} (Methods). To make a connection to the flat-band lattice model of interest, in Fig. 1a we label the 12 atomic levels that are used, and in Fig. 1b,c we depict how these states relate to the unit cells and sublattice sites of the rhombic or diamond lattice^18,20,24,41^. This well-studied lattice possesses a unit cell with three sublattice sites, which we label a, b and c. Our 12-state implementation of periodic boundary conditions thus contains four unit cells, which we label by an index *m* ∈ [0, 3]. By controlling the relative phases of the applied microwave tones, we control the values of a uniform *U*(1) abelian flux *ϕ* that penetrates each rhombic plaquette as well as the ‘twist phase’ *θ* associated with twisted boundary conditions (TBCs)^39^, shown in Fig. 1b,c.

We implemented the rhombic lattice Hamiltonian:

1 $$\begin{array}{l}\hat{H}=\frac{\varOmega }{2}\sum _{m\in [0,3]}\left[\operatorname{e}^{\mathrm{i}{\phi }_{\mathrm{ab},m}^{(1)}}{\hat{b}}_{m}^{\dagger }{\hat{a}}_{m}+\operatorname{e}^{\mathrm{i}{\phi }_{\mathrm{ca},m}^{(1)}}{\hat{a}}_{m}^{\dagger }{\hat{c}}_{m}\right.\\\left.\quad\,+\operatorname{e}^{\mathrm{i}{\phi }_{\mathrm{ba},m}^{(2)}}{\hat{a}}_{m+1}^{\dagger }{\hat{b}}_{m}+\operatorname{e}^{\mathrm{i}{\phi }_{\mathrm{ac},m}^{(2)}}{\hat{c}}_{m}^{\dagger }{\hat{a}}_{m+1}\right]+{\rm{h.c.}}\end{array}$$

with ${\hat{a}}_{4}={\hat{a}}_{0}$ for TBCs. Here, ${\hat{\alpha}}_{m}^{\dagger} ({\hat{\alpha}}_{m})$ is the creation (annihilation) operator for sublattice site *α* ∈ {*a*,*b*,*c*} of unit cell *m* and the *ϕ* terms relate to the phases of specific hopping elements. Specifically, ${\phi_{\mathrm{\alpha\beta},m}^{(x)}}$ relates to the phase acquired when hopping from sublattice site *β* to *α*, within unit cell *m* when *x* = 1 and between adjacent unit cells when *x* = 2. The in-diamond plaquette phase *ϕ* and the twist phase *θ* were set by letting ${\phi }_{\mathrm{ab},0}^{(1)}=\theta$, ${\phi }_{\mathrm{ca},0}^{(1)}=\phi -\theta$, ${\phi }_{\mathrm{ab},2}^{(1)}=\phi$, ${\phi }_{\mathrm{ac},1}^{(2)}=\phi$ and ${\phi }_{\mathrm{ba},3}^{(2)}=\phi$, whereas the other phases were set to zero. The tunnelling energies were set to a uniform value of *Ω*/2 by calibrating and controlling the megahertz-scale state-to-state Rabi rates (Supplementary Fig. 1). The uniform but tunable flux values *ϕ* were calibrated by measuring the single-atom dynamics for isolated plaquettes. Finally, the twist angle *θ* was controlled by the intracell tunnelling phases of the first unit cell, as indicated by the dashed lines in Fig. 1a–c. TBCs^39^ allow for the exploration of effectively large systems with translation invariance, by using the twist phase *θ* to encode the phase accumulated by Bloch states with quasimomentum *k*. This correspondence is depicted in Fig. 1d,e, which reveals the dispersion of eigenenergies with *k* and *θ* when *ϕ* = 0, contrasted with the flat response of all bands due to AB caging when *ϕ* = π. The energy dispersion of the (four-unit-cell) TBC lattice versus *θ* (Fig. 1e) is just the fourfold-folded version of the infinite lattice dispersion versus *k* (Fig. 1d).

**Fig. 2:**
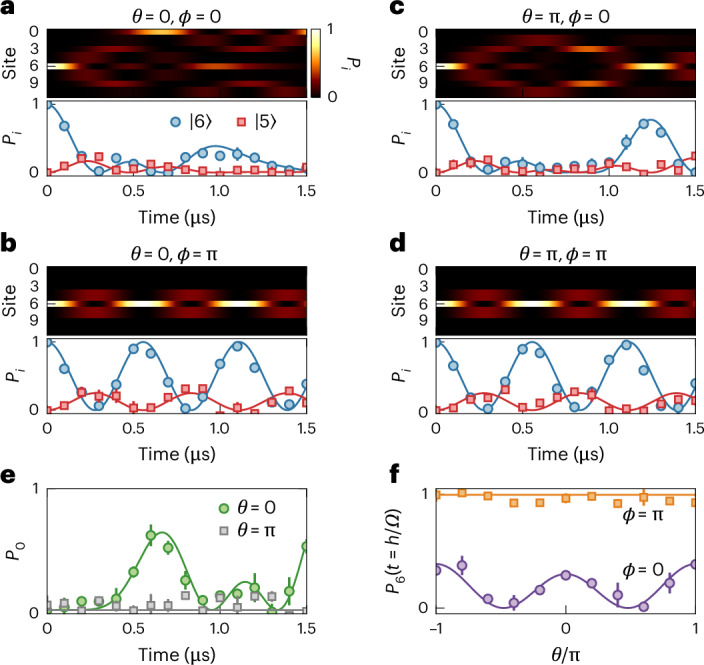
Phase-dependent single-atom caging dynamics on a synthetic rhombic lattice. **a**–**d**, Top, time evolution of the site populations (Rydberg state) from numerical simulations of equation (1) under different combinations of the twist phase *θ* and plaquette phase *ϕ*: (**a**) *θ* = 0, *ϕ* = 0, (**b**) *θ* = 0, *ϕ* = π, (**c**) *θ* = π, *ϕ* = 0 and (**d**) *θ* = π, *ϕ* = π. Bottom, experimentally measured population dynamics (corrected for SPAM errors; Methods) for states $\left\vert 5\right\rangle$ (red squares) and $\left\vert 6\right\rangle$ (blue circles) under the same phase combinations. Coupling strength *Ω*/*h* = 0.90(2) MHz. **e**, Time evolution of the population in state $\left\vert 0\right\rangle$ for *θ* = 0 (green circles) and π (grey squares), for plaquette phase *ϕ* = 0. **f**, Population in the initial state $\left\vert 6\right\rangle$ after dynamics for a time *t* = *h*/*Ω* ≈ 1.1 μs as a function of the twist phase *θ*, for *ϕ* = 0 (purple circles) and π (orange squares). Solid lines are numerical simulations with the ideal tight-binding Hamiltonian. Error bars are the standard error from several independent datasets.

The results of experimental single-atom dynamics in the twisted rhombic lattice are presented in Fig. 2. Starting with atoms prepared in state $\left\vert 6\right\rangle$, the microwave-driven dynamics reveal our flux-based control of single-atom AB caging. Figure 2a–d shows the full set of simulated state dynamics, along with the measured and simulated dynamics for the initialized state ($\left\vert 6\right\rangle$, blue circles) and a neighbouring state ($\left\vert 5\right\rangle$, red squares). The measured population data have been renormalized (scaled) based on calibration measurements to correct for state preparation and measurement (SPAM) errors (Methods). The dynamics of more states are presented in Supplementary Fig. 2.

The dynamics under uncaged (*ϕ* = 0) and caged (*ϕ* = π) conditions are contrasted in Fig. 2a,b for zero twist phase. In the absence of caging (*ϕ* = 0), the population spread out from the initial unit cell. In contrast, under AB caging (*ϕ* = π), the population oscillated between state $\left\vert 6\right\rangle$ and its four neighbouring states. The initial population projected onto just three nearby CLSs that beat against each other leading to short-range oscillatory dynamics but no large-scale delocalization. These two trends are reflected in the measured population dynamics for states $\left\vert 5\right\rangle$ and $\left\vert 6\right\rangle$ in Fig. 2a,b, which are in good agreement with the simulations and show the emergence of large, regular population revivals under caging.

Figure 2c,d presents corresponding simulations and state population dynamics but for twist phase *θ* = π. Although the dynamics under caging in Fig. 2d are identical to those in Fig. 2b, the change of twist phase from 0 to π led to notable modifications of the uncaged dynamics in Fig. 2c. Microscopically, a π twist phase around the periodic boundary structure of Fig. 1c can be understood as having caused another, larger-scale, caging condition, wherein the population starting at state $\left\vert 6\right\rangle$ was forbidden from reaching the opposing site $\left\vert 0\right\rangle$ due to destructive interference. This resulted in the enhanced revival at state $\left\vert 6\right\rangle$ after a time ~1.2 μs, as seen in Fig. 2c.

We probed this larger-scale caging condition more directly (Fig. 2e) by measuring the state $\left\vert 0\right\rangle$ population dynamics under a twist phase of 0 (green circles) and π (grey squares). Indeed, following initialization at $\left\vert 6\right\rangle$, we found that the population reached $\left\vert 0\right\rangle$, in good agreement with the theory (equation (1)) under zero twist phase, whereas very little of the population reached $\left\vert 0\right\rangle$ for *θ* = π. In Fig. 2f, we probe another key feature of the twist phase *θ*, namely the independence of the non-equilibrium dynamics on *θ* under AB caging conditions. We specifically measured the population remaining at the initialized state $\left\vert 6\right\rangle$ after a time of ~1.1 μs, corresponding to two oscillations with caging (*t* = *h*/*Ω*). We found that the dynamics were dependent on *θ* for *ϕ* = 0 (purple circles), reflecting the dispersive energy bands under the non-caging conditions in Fig. 1d,e, whereas the measurements were essentially independent of *θ* for the caging condition (*ϕ* = π, orange squares), consistent with the all-flat-bands condition.

## Interaction-induced breakdown of caging

More generally, the observed AB caging and flat bands result from kinetic frustration. Generically, perturbations strong enough to mix the bands can disrupt frustration and induce delocalization^25^. Recently, the onset of transport under the addition of strong disorder has been observed in flat-band lattices^18,19^, which is related to inverse Anderson localization. More intriguingly, it has been predicted that interparticle interactions alone can lead to the emergent breakdown of flat-band localization^24^. Although nonlinear modifications to flat lattice bands have recently been measured in bosonic quantum gases^15,16^, the predicted delocalization of particles due to strong interactions has not yet been realized.

**Fig. 3:**
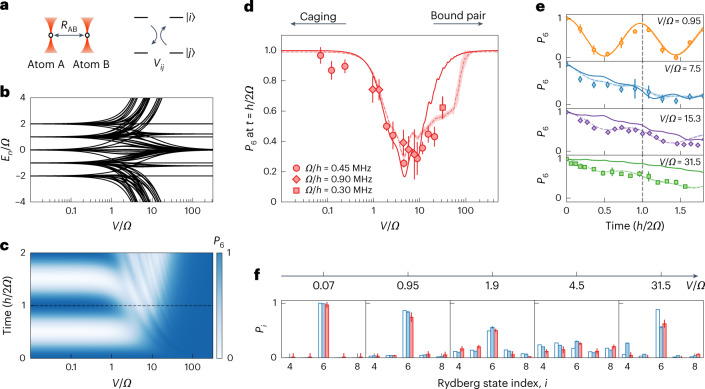
Breakdown of AB caging by interactions and the crossover to bound-pair dynamics. **a**, Atom pair A and B prepared in tweezers with a spatial separation of *R*_AB_. The dipolar exchange interaction for states $\left\vert i\right\rangle$ and $\left\vert\; j\right\rangle$ is *V*_*ij*_. We scaled all interactions to *V* = *V*_67_ with calculated *C*_3_ coefficients (Supplementary Information). **b**, Eigenenergy distribution for atom pairs in the 12-state rhombic lattice (with *ϕ* = π and *θ* = 0) under different interaction-to-coupling ratios *V*/*Ω*. Here *n* is the eigenstate index. **c**, Time evolution of the population in $\left\vert 6\right\rangle$, *P*_6_, for different *V*/*Ω* ratios from ideal numerical simulations. The dashed line indicates the single-atom revival time. **d**, Measured crossovers from caging to delocalization to pairs with reduced mobility. **e**, Experimentally measured time evolution of the SPAM-corrected population in $\left\vert 6\right\rangle$ for different *V*/*Ω*. The vertical grey dashed line again corresponds to the single-atom revival time. **f**, Population distributions for the central five sites at the first revival time *t* = *h*/2*Ω* for different *V*/*Ω*. The red and white bars correspond to experimental measurements and ideal numerical simulations, respectively. The blue bars indicate the full numerical simulations that include all interactions (including state-changing terms), SPAM errors and finite-temperature effects. Solid lines in **d** and **e** are simulations based on equation (1) with resonant flip-flop interactions, whereas the dashed lines and their shaded confidence intervals are Monte Carlo simulations that also include state-changing interaction terms and thermal variations of *R*_AB_, along with contributions from single-atom dynamics due to state preparation infidelity. All error bars are the standard error of several independent datasets.

Using pairs of Rydberg atoms with dipole–dipole interactions, we next explored the dynamics of strongly interacting pairs in the same flat-band lattice from Figs. 1 and 2. As depicted in Fig. 3a, we studied pairs of atoms prepared in an optical tweezer array, labelled A and B and spaced by a tunable distance *R*_AB_ > 4 μm, relating to tunable megahertz-scale interactions. The atoms primarily interacted through a resonant dipolar exchange^37^, which is related to the anticorrelated hopping of the A and B Rydberg electrons along the synthetic lattice^29,38^, characterized by a rate ${V}_{ij}\propto {C}_{3}^{ij}/2{R}_{{\rm{AB}}}^{3}$ for the transition ${\left\vert i\right\rangle }_{{\rm{A}}}{\left\vert\; j\right\rangle }_{{\rm{B}}}\leftrightarrow {\left\vert\; j\right\rangle }_{{\rm{A}}}{\left\vert i\right\rangle }_{{\rm{B}}}$ (with ${C}_{3}^{ij}$ the state-dependent *C*_3_ coefficient and *i* and *j* the corresponding Rydberg state indices; see Supplementary Tables I and II for more details). These interactions lack translational symmetry along the synthetic dimension due to the dependence of the exchange rates on the participating Rydberg levels (Supplementary Information). For simplicity, we characterized the A–B interactions by a single scale *V* relating to the rate *V*_67_ for ${\left\vert 6\right\rangle }_{{\rm{A}}}{\left\vert 7\right\rangle }_{{\rm{B}}}\leftrightarrow {\left\vert 7\right\rangle }_{{\rm{A}}}{\left\vert 6\right\rangle }_{{\rm{B}}}$ exchange, but note that our simulations did account for the variation of the individual resonant dipolar interaction terms (see the Supplementary Information for more details of the numerical simulations and for a comparison to a model that assumes uniform interaction terms).

For moderate strengths, despite their structure along the synthetic dimension, we expected that the interactions would generically disrupt the AB caging and induce transport. This is reflected in the energy spectrum in Fig. 3b, where for *V*/*Ω* ≈ 1, the flat, isolated energy bands became strongly mixed. The resulting reorganization of the system eigenstates would also be reflected in the non-equilibrium dynamics. Figure 3c shows the simulated dynamics of the mean state $\left\vert 6\right\rangle$ population, *P*_6_, as a function of *V*/*Ω*, for an initial product state ${\left\vert 6\right\rangle }_{{\rm{A}}}{\left\vert 6\right\rangle }_{{\rm{B}}}$. Like the energy spectrum, three regimes were expected: robust revivals due to AB caging for *V*/*Ω* ≲ 1, a decay due to delocalization dynamics for 1 ≲ *V*/*Ω* ≲ 20 and a freeze-out of *P*_6_ dynamics for very large *V*/*Ω* due to the inhibition of hopping by strong, nearly random interactions (Supplementary Information)^42^. We specifically note that whereas strong interactions alone should inhibit transport on short timescales, the unique structure of interactions along the Rydberg synthetic dimension (with substantial variations of the dipolar exchange terms for different state pairs that is proportional to their *C*_3_ coefficients as listed in Supplementary Tables I and II) can be expected to result in an actual localization due to interaction disorder^42^.

For Fig. 3d, we experimentally measured *P*_6_ for interacting atom pairs at the first single-atom revival time (*t* = *ℏ*/2*Ω*, dashed line in Fig. 3c), using several values of the spacing *R*_AB_ and the tunnelling strength to vary *V*/*Ω* by more than two orders of magnitude. We observed the expected disruption of AB caging for intermediate interactions, in good agreement with the ideal simulations (solid line) based on equation (1) and resonant dipolar exchange interactions (ignoring non-resonant, state-changing exchange terms; Supplementary Information). For strong interactions, we found that the measured *P*_6_ rises back up, in qualitative agreement with the expectations based on Figs. 3b,c. For the largest interactions (*V*/*Ω* ≳ 10), the data began to lose agreement with the idealized description of resonant exchange interactions that conserve the net populations (A and B combined) of each Rydberg level. Better agreement was found when we accounted for the expected contributions from dipolar interaction terms that interconvert spin and orbital angular momentum^37^, represented by the ‘full simulation’ dashed curve in Fig. 3d. With the inclusion of the state-changing dipolar interactions, which can be thought of as both enabling off-resonant (pairwise) transitions to other states and introducing perturbative corrections to the synthetic lattice site energies, a complete freeze-out of the *P*_6_ dynamics was not expected until inaccessibly large interactions (*V*/*Ω* ≳ 100). The dashed lines also account for minor sources of parameter uncertainty, that is, from calibration uncertainty and the thermal spread of interactions (Supplementary Information).

For broader context on the Rydberg lattice platform, note that control of the parameters in our study was mainly limited by technical considerations. The finite Rydberg lifetime limited our evolution timescales, and correspondingly how small we could set *Ω* (see ref. ^31^ for longer-time dynamics using higher-*n* Rydberg states). In the other limit, when choosing to increase the scales of *V* and *Ω*, we were limited by the relatively small Zeeman energy splittings between various Rydberg *m*_*j*_ levels. For large *Ω* or *V*, these small energy separations resulted in the off-resonant driving of microwave transitions or the activation of non-resonant dipolar interactions, respectively. In a larger magnetic bias field, the upper ranges of the *Ω* and *V* control could reasonably be increased by an order of magnitude.

Figure 3e plots traces of the measured dynamics for a few representative values of *V*/*Ω*. There is good agreement with the expected pair dynamics (dashed curves). We identified two main sources of disagreement between the data and the simple idealized model (solid lines). For intermediate *V*/*Ω* (*V*/*Ω* = 7.5), the disagreement arose mainly from the non-negligible contribution of single atoms. With a 92% Rydberg state preparation fidelity (combination of the efficiencies of optical pumping and the stimulated Raman adiabatic passage), roughly 15% of our ‘pair’ data contained only a single Rydberg atom. This was accounted for in the dashed curve by weighting the expected dynamics of singles and pairs. For large interactions, *V*/*Ω* ≳ 10, the non-resonant state-changing interactions became the dominant source of disagreement. Such dipolar terms^37^, which did not conserve the net populations of individual Rydberg states, were energetically suppressed in the presence of a magnetic bias field but could still have affected the dynamics when *V* was large.

Figure 3f separately displays the population of several different Rydberg states as a further probe of the predicted interaction-induced breakdown of AB caging. After an evolution for *t* = *ℏ*/2*Ω*, we read out the population at different Rydberg levels by performing state-swapping microwave transitions before the optical de-excitation of 42*S*_1/2,1/2_. We plot histograms of the populations for states $\left\vert 4\right\rangle$ to $\left\vert 8\right\rangle$ (the initialized state and its neighbours) and compare the measured populations (red bars) to those predicted by the full (blue) and ideal (white) simulations. In general, we found good evidence for a sizable population residing on state $\left\vert 6\right\rangle$ for both small and large *V*/*Ω*, with the population spread across the set of measured states for intermediate *V*/*Ω* values. Although we focused on the delocalization of just two interacting particles, the Rydberg synthetic dimensions can be extended to hundreds of atoms^43^ and many dozens of states, so that this approach is a versatile complement to explorations of correlated quantum walks with neutral atoms^44,45^ and photons^46^.

## Emergent magnetism within interacting flat bands

**Fig. 4:**
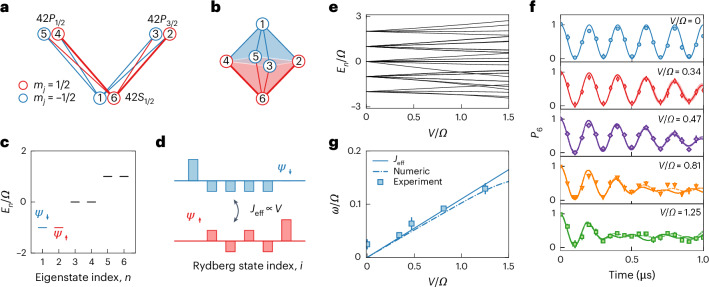
Emergent magnetism in a flat-band π-flux rhombic bipyramid. **a**,**b**, Six Rydberg levels are coupled by eight microwave tones (**a**) to generate the bipyramid structure (**b**). The two connections for $\left\vert 6\right\rangle \leftrightarrow \left\vert 2\right\rangle$ and $\left\vert 6\right\rangle \leftrightarrow \left\vert 4\right\rangle$, indicated by bold lines, have a π phase shift relative to the other transitions. **c**, Doubly degenerate eigenspectrum of the non-interacting six-state system. We denote the degenerate pairs of eigenstates by effective pseudospins *Ψ*_*↓*_ (blue) and *Ψ*_*↑*_ (red), shown for the lowest two states. **d**, Representation of the lowest degenerate single-atom eigenstates *Ψ*_*↑*_ (red) and *Ψ*_*↓*_ (blue). In the presence of a weak dipolar exchange (*V* ≪ *Ω*), the emergent magnetic interactions *J*_eff_ are proportional to *V* (scaled to *V* = *V*_62_; Supplementary Information). **e**, Atom pair eigenenergies for increasing *V*/*Ω*. **f**, Time evolution of the SPAM-corrected mean population in $\left\vert 6\right\rangle$ for different *V*/*Ω* ratios. The coupling strength *Ω*/*h* = 2.50(4) MHz. The interaction strength *V* was varied by changing the atomic separation. The solid lines are numerical simulations with experimental parameters. The dashed lines and shaded regions are numerical simulations that consider both the finite-temperature spread of interactions and the Rydberg state preparation infidelity. **g**, Short-time beating frequency *ω* versus the interaction strength, found by fitting the experimental measurements over the first 0.5 μs to the function ${P}_{6}(t)=a+b\cos (2\omega t/\hslash )\cos (2\varOmega t/\hslash )$. The solid line is the analytical prediction *J*_eff_ based on the emergent magnetism in the {*Ψ*_*↓*_, *Ψ*_*↑*_} subspace (Supplementary Information). The dotted dashed line is a fit to the numerical results in **f** over the same time and with the same function. All error bars are standard errors from several experimental measurements.

Our observation of the interaction-induced breakdown of AB caging in Fig. 3 occurred in the *V* > *Ω* regime, where pairwise interactions were so large that they mixed the bands and destroyed the flat-band localization. In contrast, when *V* < *Ω*, weak interactions did not directly mix the bands but acted as a first-order perturbation to the physics of the individual, isolated bands. This regime resulted in rich, emergent physics driven by interactions. In generic flat bands occupied by short-range-interacting particles, one encounters emergent long-range interactions and phenomena like charge-density-wave ordering^47^. More exotic phenomena have been encountered in topological flat bands^48^, including analogues of the fractional quantum Hall effect in lattice systems^8^. In Fig. 4, we use a simple flat-band structure to explore the emergent magnetism of localized flat-band states.

Figure 4a,b depicts the six Rydberg levels we used and the geometric representation of the engineered flat-band structure. Solid lines denote the hopping links. A π hopping phase was applied to the two thick lines ($\left\vert 4\right\rangle \leftrightarrow \left\vert 6\right\rangle$ and $\left\vert 6\right\rangle \leftrightarrow \left\vert 2\right\rangle$). The resulting structure is a three-dimensional rhombic bipyramid that has a π flux penetrating each of its four surfaces, which can be thought to result from an enclosed magnetic monopole. This leads to an AB caging, with three degenerate pairs of CLSs. This is exemplified in Fig. 4c,d, where we label the ground pair of degenerate states as *Ψ*_*↓*_ and *Ψ*_*↑*_. The result of weak interactions between neighbouring Rydberg atoms (referred to again as A and B) is an emergent spin-1/2 quantum magnetism of these degenerate pseudospin state pairs (Supplementary Information), reflected in the linear splitting of two-atom eigenstates in Fig. 4e with increasing interactions.

In the idealized picture of uniform interactions between nearest synthetic neighbours (*V*_6*j*_ = *V*_1*j*_ = *V* for *j* ∈ [2, 5]), the low-energy physics is effectively described by the spin model ${J}_{{{XX}}}({\sigma }_{{\rm{A}}}^{\;x}{\sigma }_{{\rm{B}}}^{\;x}+{\sigma }_{{\rm{A}}}^{\;y}{\sigma }_{{\rm{B}}}^{\;y})+{J}_{{{ZZ}}}({\sigma }_{{\rm{A}}}^{z}{\sigma }_{{\rm{B}}}^{z})$, where *J*_*ZZ*_ = *J*_*XX*_ = *V*/4 in the well-studied Heisenberg XXX model. Here, $\sigma^j_\alpha$ is the Pauli-*j* operator acting on atom *α* (with $j \in \{x,y,z\}$ and $\alpha \in \{A,B\}$) and the symbols *J*_*XX*_ and *J*_*ZZ*_ denote the strengths of the flip-flop ($\sigma^x \sigma^x + \sigma^y \sigma^y$) and Ising ($\sigma^z \sigma^z$) interactions. Considering the state-specific form of the *V*_*ij*_ in our system, the actual description includes another spin–spin interaction term, ${J}_{{{XZ}}}({\sigma }_{{\rm{A}}}^{\;x}{\sigma }_{{\rm{B}}}^{z}+{\sigma }_{{\rm{A}}}^{z}{\sigma }_{{\rm{B}}}^{\;x})$. With the experimental interaction terms {*V*_62_, *V*_63_, *V*_64_, *V*_65_} = {*V*, −*V*/4, *V*/2, −*V*/2} and {*V*_12_, *V*_13_, *V*_14_, *V*_15_} = {− *V*/4, *V*, *−V*/2, *V*/2} (with *V* ≡ *V*_62_), we have *J*_*XZ*_ = 3*J*_*ZZ*_ = 3*J*_*XX*_ = 9*V*/64. To probe this emergent magnetism, we simply monitored the dynamical response of atom pairs initialized to the state ${\left\vert 6\right\rangle }_{{\rm{A}}}{\left\vert 6\right\rangle }_{{\rm{B}}}$. Figure 4f shows the dynamics of the mean *P*_6_ for several interaction-to-hopping ratios *V*/*Ω*. For weak *V*/*Ω*, we observe two well-separated contributions to the dynamics: a fast oscillation and a slower beating that leads to an apparent contrast decay. The fast term, ~*Ω*, is related to the intracell dynamics that stems from the beating of the different energy bands. The low-frequency part results from the interaction-induced splitting of the individual bands and is directly related to the emergent magnetism. For our physical interactions, we expected it to scale approximately as ${J}_{{\rm{eff}}}=\sqrt{{J}_{{{XX}}}^{2}+{J}_{{{XZ}}}^{2}/2}$. Figure 4g shows the low-frequency contribution determined from the fitting, *ω*. This emergent oscillation term scales roughly linearly with *V* for small *V*/*Ω*, in good agreement with the *J*_eff_ prediction and with the prediction based on numerical simulations. It begins to deviate from a linear relation (proportional to *V*) at large *V* due to band mixing.

## Conclusion

These explorations of strongly interacting particles in flat-band lattices suggest several future extensions. Using the many states available to Rydberg synthetic lattices, it is possible to explore the related phenomena of non-abelian AB caging^49^ and caging structures with non-abelian symmetries^50,51^, as well as AB caging in higher (three or even four) dimensions. By working at higher principal quantum numbers, by using Rydberg *D* orbital states or simply by using even higher bandwidth microwave sources, it is realistic to envision the extension to lattices with over 100 synthetic sites. Extending to topological flat-band models, we may ask whether the non-local (in space) interactions can result in emergent topological order. Finally, in the context of pseudomagnetism emerging from the projection of interactions onto flat-band CLSs, higher-spin models will emerge from lattices with more unit cells (for example, a spin-3/2 model emerges from the four-cell rhombic lattice), providing a route for exploring, for example, emergent quantum Potts models^52,53^. In this context, although our current study is limited to pairs of atoms due to Rydberg state preparation infidelity, one can readily envision extending such Rydberg synthetic lattice experiments to larger one- and two-dimensional atom arrays^54^ with hundreds of atoms.

## Methods

### Microwave control system

Compared to our previous demonstration of Rydberg synthetic dimensions in atom arrays^29^, here we drive more transitions between more Rydberg levels. Most importantly, to address transitions between states with principal quantum numbers *n* = 42, 43 and 44, here we had to apply coherent microwaves over a greater span of frequencies. To address these transitions, here we modified the set-up used in ref. ^29^ to use a Tabor P9484D for the intermediate-frequency signal, achieving a bandwidth of ~9 GHz centred around a frequency of approximately 48 GHz.

### Calibrating synthetic lattice hopping amplitudes and plaquette fluxes

We calibrated the mapping between the parameters of our applied microwave spectrum and those of the effective tight-binding model, equation (1), based on the atomic response. The control of the parameters (tunnelling amplitudes, plaquette fluxes and twist phase) through the microwave tone parameters is discussed in the main text. In short, we calibrated the hopping rates by simply measuring the state-to-state Rabi dynamics. We calibrated the fluxes in each of the four primary lattice plaquettes by measuring the recurrence dynamics beginning from one of the adjoining A sublattice sites. We similarly calibrated the twist phase by measuring the dynamical response. More details of the calibration procedures are given in the Supplementary Information.

### Corrections for state preparation and read-out infidelity

As discussed in ref. ^29^, there are two limiting quantities to note: the upper and lower limits of the measured raw data. First, there is an upper ceiling that is on average equal to *P*_u_ = 0.88(1), which stems from an inefficiency of the stimulated Raman adiabatic passage and loss during release and recapture. There is also a lower baseline of the measurements, having an average value *P*_l_ = 0.21(1), which we believe stems from the decay (and subsequent recapture) of the short-lived Rydberg states (*n* ∈ {42, 43, 44}), such that the Rydberg states have some probability of appearing bright in a subsequent fluorescence detection. These infidelities limit the contrast of the state population dynamics.

We renormalized all the data to account for these known infidelities in the following way: we defined the renormalized populations *P*_*i*_ in relation to the measured bare populations ${P}_{i}^{{\rm{bare}}}$ as ${P}_{i}=({P}_{i}^{{\rm{bare}}}-{P}_{l})/({P}_{u}-{P}_{l})$. In the main text, we refer to such renormalized population measurements as being corrected for SPAM.

Note that the statistical fluctuations associated with the processes that motivate this discussed renormalization are systematically not reflected in either the error bars of the renormalized data nor the error bars of the full theory, which only accounts for the calibrated uncertainties of the control parameters and the thermal fluctuations of the interatomic separations.

### Accounting for parameter uncertainties in the numerical simulations

For our full simulations (dashed lines), we also included confidence intervals (shaded regions) that reflect our uncertainties of the calibrated system parameters as well as the shot-to-shot position variance of the finite-temperature atoms due to trap release; for details of the Monte Carlo simulations used to model our confidence intervals, see ref. ^29^. Note that the shaded confidence regions shown in Figs. 3d,e and 4f were largely determined by the spread (uncertainty) of the interaction strengths that resulted from the thermal spread of the interatomic distances.

## Online content

Any methods, additional references, Nature Portfolio reporting summaries, source data, extended data, supplementary information, acknowledgements, peer review information; details of author contributions and competing interests; and statements of data and code availability are available at 10.1038/s41567-024-02714-7.

## Supplementary information

Supplementary Information Supplementary Figs. 1–4, discussion and Tables I and II.

## Data Availability

All of the experimental data from this work are available via Zenodo at 10.5281/zenodo.13218546 (ref. ^55^).
