# Supplementary material for: Interaction-driven breakdown of Aharonov–Bohm caging in flat-band Rydberg lattices
Source: Nat Phys. 2025 Jan 10;21(2):221–7. doi: 10.1038/s41567-024-02714-7 (PMC11825368; doi:10.1038/s41567-024-02714-7)
Supplement: Supplementary file 1 — Supplementary Figs. 1–4, discussion and Tables I and II. [file 41567_2024_2714_MOESM1_ESM.pdf]

nature physics

Supplementary information https://doi.org/10.1038/s41567-024-02714-7

Interaction-driven breakdown of Aharonov–Bohm caging in flat-band Rydberg lattices

In the format provided by the authors and unedited

Supplementary Information for "Interaction-driven breakdown of Aharonov–Bohm caging in flat-band Rydberg lattices"

ADDITIONAL EXPERIMENTAL DETAILS

Details on the calibrations of synthetic lattice hopping amplitudes and plaquette fluxes

Figure S1(a) shows an example calibration of state-to-state hopping rates. To measure the Rabi frequency between state $|i\rangle$ and $|j\rangle$, we first apply a series of high-fidelity π-pulses to transfer all population from initially prepared state $|6\rangle$ to state $|i\rangle$, then measure the $|i\rangle \leftrightarrow |j\rangle$ oscillation by varying the single-tone microwave pulse duration followed by another series of π-pulses (from $|i\rangle$ back to $|6\rangle$) and the detection "pulse" (975 nm laser, depumping from $|6\rangle$ to the ground $4S_{1/2}$ state for fluorescence imaging) [1]. We vary the IF amplitudes for each specific frequency tone to make the Rabi frequencies for all relevant transitions nearly uniform (to a value we refer to as Ω/h). Our microwave generation system works within the linear response regime, so we can simply scale the global output amplitude of the IF generator to globally change the value of Ω after calibration. Additionally, we note that the calibration of the individual links in the absence of other applied tones yields results that are consistent with the response in the presence of all tones (i.e., corrections due to off-resonant coupling terms from the other drives are small and ignorable).

Figure S1(b) shows how we calibrate the in-diamond, or plaquette, flux ϕ for the $|6\rangle \rightarrow |7\rangle \rightarrow |9\rangle \rightarrow |8\rangle \rightarrow |6\rangle$ plaquette (see Fig. 1 of the main text for the state configuration mapping). We let the frequency tone (sine wave) in the IF generator output for the $|i\rangle \rightarrow |j\rangle$ transition take a phase of φ_{ij}. For this plaquette, we let $\varphi_{79} = \varphi_{98} = \varphi_{86} = 0$ and scan φ_{67} from 0 to 2π to check the in-diamond phase-dependent dynamics (all these phases refer to the initial phase values at the source). Similar to Ref. [1], after an evolution time of $t = h/\Omega$, the population in $|6\rangle$ has a peaked recurrence for zero in-diamond phase. As shown in Fig. S1(b), the peak location at $\varphi_{67} = 1.16(1)\pi$ indicates the in-diamond phase $\phi = 0$ at the atoms. We then simply account for the the calibrated offset phase $\varphi_c = 1.16\pi$ in the IF output program. We find that this calibrated offset phase value is very stable in our day-to-day measurements. For other plaquettes, we repeat a similar calibration procedure based on population recurrence (using a series of π pulses to initiate population at, and read out population from, the states at the corner of those plaquettes).

After the calibrations of the state-to-state coupling strengths and each in-diamond flux, we address the twist phase θ by simply scanning the extra additional phases $-\theta_{\text{ex}}$ on both the $|1\rangle \rightarrow |0\rangle$ and $|2\rangle \rightarrow |0\rangle$ transitions (see the main text - these phases cancel in their contributions to the $m = 0$ plaquette flux, but contribute to the global twist phase). We initiate population at state $|6\rangle$ and measure the remaining population, P_6, after the system evolves $t = \hbar/\Omega \sim 1.1$ μs) with $\Omega/h = 0.9(2)$ MHz. From the theoretical simulations, P_6 peaks at twist phase values of both 0 and π at this time (when $\phi = 0$, under the non-caged condition, see solid curve Fig. 2(f) of the main text). As shown in Fig. S1(c), we fit the measured P_6 with a set of Gaussian peaks separated by π phase and obtain that $\theta_{\text{ex}} = \theta_c = -0.60(1)\pi$ corresponds to the global twist phase of $\theta = 0$ (the determination of which peak corresponds to 0 and which to π is made by the value of P_6 at a slightly later time, $\sim$1.2 μs). For the experimental implementation, we directly account

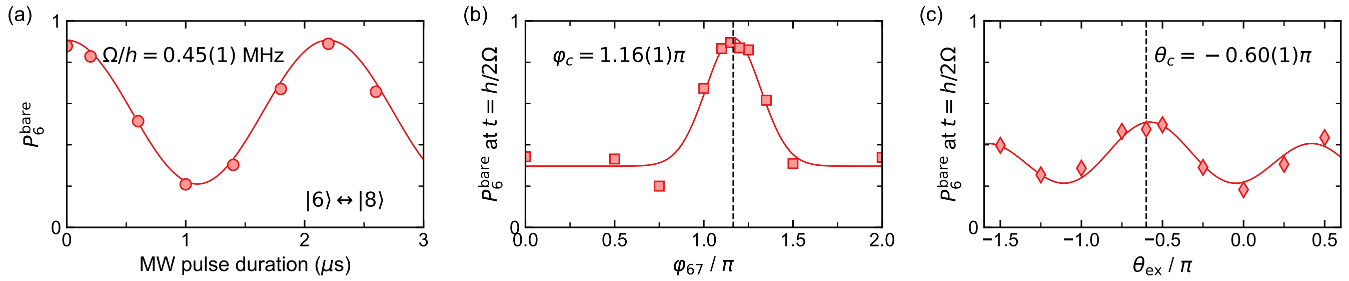

FIG. S1. **Calibration of state-to-state coupling strength Ω, in-diamond flux ϕ, and twist phase θ. (a)** Rabi oscillation for $|6\rangle \leftrightarrow |8\rangle$ transition. Fitting with a sine function (solid line) to the experimental data (circles) gives the coupling strength is $\Omega/h = 0.45(1)$ MHz. **(b)** Population in $|6\rangle$ after an evolution time of $t = h/2\Omega$ (~ 1.1 μs) versus the relative source phase φ_{67} for the $|6\rangle \rightarrow |7\rangle$ tone in the IF generator output signal. The Gaussian fit (solid line) gives the peak location $\varphi_c = 1.16(1)\pi$, which corresponds to zero in-diamond flux $\phi = 0$. **(c)** Population in $|6\rangle$ after an evolution time of $t = h/\Omega$ (~ 1.1 μs, $\Omega/h = 0.90(2)$ MHz) with in-diamond flux $\phi = 0$ versus the additional phase θ_{ex} for $|1\rangle \rightarrow |0\rangle$ and $|2\rangle \rightarrow |0\rangle$ transitions. By fitting the measured data with a 3-peak Gaussian function (peaks at θ_c and $\theta_c \pm \pi$), we get the calibrated twisted phase $\theta_c = -0.60(1)\pi$ that corresponds to zero twist phase.

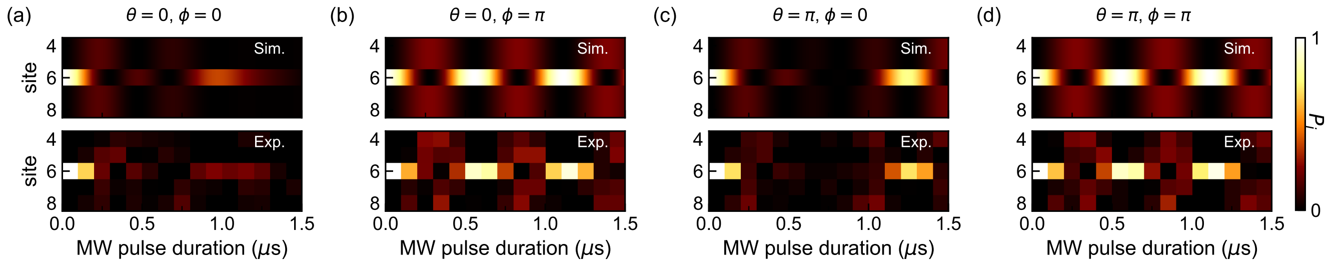

FIG. S2. **Additional experimental data for the single-atom population dynamics of main-text Fig. 2, under different phase (θ and ϕ) combinations, showing the simulated dynamics (top) and measured population dynamics (bottom) for each state $|i\rangle$ ($4 \le i \le 8$).** **(a)** Dynamics for $\phi = 0$ and $\theta = 0$. **(b)** Dynamics for $\phi = \pi$ and $\theta = 0$. **(c)** Dynamics for $\phi = 0$ and $\theta = \pi$. **(d)** Dynamics for $\phi = \pi$ and $\theta = \pi$.

for a calibration parameter $\theta_c = -0.60\pi$ in the IF output program to achieve a desired θ value at the atoms.

Additional state dynamics

In the context of single-atom AB caging, we primarily present data for states $|5\rangle$ and $|6\rangle$ in the main text. However, we measured population dynamics for all the states in the vicinity of the initially populated $|6\rangle$ - namely, states $|4\rangle$, $|5\rangle$, $|6\rangle$, $|7\rangle$, and $|8\rangle$. Figure S2 presents the extended dynamics data set for all of these state populations, for the same phase combinations of ϕ and θ as presented in Fig. 2 of the main text. Overall, all the state population dynamics are in good agreement with the theory predictions. For the cases of AB caging [$\phi = \pi$, panels (b,d)], where initialization at state $|6\rangle$ should lead simply to a breathing dynamics between occupation of the $|6\rangle$ site and equal-weight occupation at the states $|4\rangle$, $|5\rangle$, $|7\rangle$, and $|8\rangle$, we indeed observe nearly identical dynamics at each of these outer sites of the CLS.

ADDITIONAL THEORETICAL DETAILS

Modeling the dipolar interactions

In the main text we work with two different level structures: a 12-level twisted rhombic lattice (see Fig. 1) and a 6-level bipyramid (see Fig. 4). As discussed in [1], the full interaction Hamiltonian for a pair of atoms (A and B) is

$$H_{\mathrm{int}} = \sum_{i,j} \sum_{i',j'} e^{i\Delta_{ij}^{i'j'} t} V_{iji'j'} \, |i\rangle_{\mathrm{A}} \langle j| \otimes |j'\rangle_{\mathrm{B}} \langle i'| + \mathrm{h.c.} \,, \tag{S1}$$

with $V_{iji'j'}$ the dipolar interaction strength (labelled as V_{ij} when $i = i', j = j'$ below) and $\hbar\Delta_{ij}^{i'j'}$ the energy gap between state pairs $|i\rangle_{\mathrm{A}} |j'\rangle_{\mathrm{B}}$ and $|i'\rangle_{\mathrm{B}} |j\rangle_{\mathrm{A}}$.

In the weak interaction regime with large interatomic distance, i.e., $V_{iji'j'} \ll |\Delta_{ij}^{i'j'}|$, the non-resonant state-changing interaction terms only slightly affect the dynamics. We can thus largely neglect such terms and only consider the resonant state-conserving terms in "ideal" numerical simulations. Table SI lists all the relevant C_3 coefficients for resonant dipolar exchange interactions in both systems (the 12-state and 6-state lattices). We have previously calibrated the interaction strength for state pair $|6\rangle \leftrightarrow |7\rangle$ as $V_{67} = h \times 0.86(2)$ MHz, based on a 2 MHz separation between the frequency tones driving the acousto-optic deflector (AOD) used to create our optical tweezer pattern (corresponding to ~ 9.6 μm for the interatomic distance) [1]. In our experiment, to obtain different V/Ω ratios, we vary the interaction strength by directly changing the frequency gap of the tones driving the AOD or we vary the microwave-driven hopping rates by globally changing the microwave coupling strength. For numerical simulations, we scale the interaction strengths $V_{ij} \propto C_3^{ij}/2R_{\mathrm{AB}}^3$ with the calculated C_3^{ij} coefficients and the interatomic distance R_{AB} based on the calibrated V_{67} value.

For strong interactions with relatively small interatomic distances (i.e., for rather large V/Ω ratios), the state-changing terms largely account for the differences between the ideal numerical simulations and experimental measurements in the main text Fig. 3(e). To note, since the ratio $V/\Omega \lesssim 1$ for all the data relating to the 3D bipyramid structure (main text Fig. 4), and the state-changing transitions do not play a significant role in that case, here we focus

**Twisted rhombic lattice**			
$\|i\rangle$ $\|j\rangle$	C_3	$\|i\rangle$ $\|j\rangle$	C_3
$\|0\rangle$ $\|1\rangle$	-355.6	$\|6\rangle$ $\|4\rangle$	-375.6
$\|0\rangle$ $\|2\rangle$	-705.0	$\|6\rangle$ $\|5\rangle$	-756.4
$\|0\rangle$ $\|10\rangle$	1422.3	$\|6\rangle$ $\|7\rangle$	1502.4
$\|0\rangle$ $\|11\rangle$	705.0	$\|6\rangle$ $\|8\rangle$	756.4
$\|3\rangle$ $\|1\rangle$	-414.3	$\|9\rangle$ $\|4\rangle$	1289.5
$\|3\rangle$ $\|2\rangle$	-834.4	$\|9\rangle$ $\|5\rangle$	639.1
$\|3\rangle$ $\|10\rangle$	1567.3	$\|9\rangle$ $\|7\rangle$	-322.4
$\|3\rangle$ $\|11\rangle$	834.4	$\|9\rangle$ $\|8\rangle$	-639.1
$\|3\rangle$ $\|4\rangle$	-322.4	$\|9\rangle$ $\|1\rangle$	1657.3
$\|3\rangle$ $\|5\rangle$	-639.1	$\|9\rangle$ $\|2\rangle$	834.4
$\|3\rangle$ $\|7\rangle$	1289.5	$\|9\rangle$ $\|10\rangle$	-414.3
$\|3\rangle$ $\|8\rangle$	639.1	$\|9\rangle$ $\|11\rangle$	-834.4
**Bipyramid structure**			
$\|i\rangle$ $\|j\rangle$	C_3	$\|i\rangle$ $\|j\rangle$	C_3
$\|1\rangle$ $\|2\rangle$	-375.6	$\|6\rangle$ $\|2\rangle$	1502.4
$\|1\rangle$ $\|3\rangle$	1502.4	$\|6\rangle$ $\|3\rangle$	-375.6
$\|1\rangle$ $\|4\rangle$	-756.4	$\|6\rangle$ $\|4\rangle$	756.4
$\|1\rangle$ $\|5\rangle$	756.4	$\|6\rangle$ $\|5\rangle$	-756.4

TABLE SI. **Calculated C_3 coefficients (units of MHz μm^3) for the resonant dipolar exchange interaction terms.** The table lists the relevant interaction terms for both structures (12-state lattice and 6-state bipyramind) described in the main text.

**Non-resonant state changing terms**								
$\|i\rangle$ $\|j\rangle$ $\|i'\rangle$ $\|j'\rangle$	$\Delta_{ij}^{i'j'}$	C_3	$\|i\rangle$ $\|j\rangle$ $\|i'\rangle$ $\|j'\rangle$	$\Delta_{ij}^{i'j'}$	C_3	$\|i\rangle$ $\|j\rangle$ $\|i'\rangle$ $\|j'\rangle$	$\Delta_{ij}^{i'j'}$	C_3
$\|6\rangle$ $\|4\rangle$ $\|15\rangle$ $\|17\rangle$	25	-650.5	$\|6\rangle$ $\|7\rangle$ $\|4\rangle$ $\|17\rangle$	25	1502.4	$\|6\rangle$ $\|16\rangle$ $\|7\rangle$ $\|17\rangle$	25	-650.5
$\|6\rangle$ $\|4\rangle$ $\|7\rangle$ $\|17\rangle$	125	1126.8	$\|6\rangle$ $\|16\rangle$ $\|15\rangle$ $\|17\rangle$	-75	3380.3	$\|6\rangle$ $\|16\rangle$ $\|4\rangle$ $\|6\rangle$	-100	1951.6
$\|17\rangle$ $\|15\rangle$ $\|7\rangle$ $\|17\rangle$	-100	1951.6	$\|6\rangle$ $\|8\rangle$ $\|5\rangle$ $\|17\rangle$	50	-756.4	$\|17\rangle$ $\|8\rangle$ $\|5\rangle$ $\|6\rangle$	-100	-2269.1
$\|3\rangle$ $\|4\rangle$ $\|15\rangle$ $\|9\rangle$	25	-558.4	$\|3\rangle$ $\|7\rangle$ $\|4\rangle$ $\|9\rangle$	25	1289.5	$\|3\rangle$ $\|16\rangle$ $\|7\rangle$ $\|9\rangle$	25	-558.4
$\|3\rangle$ $\|4\rangle$ $\|7\rangle$ $\|9\rangle$	125	967.1	$\|3\rangle$ $\|16\rangle$ $\|15\rangle$ $\|9\rangle$	-75	2901.4	$\|3\rangle$ $\|16\rangle$ $\|4\rangle$ $\|3\rangle$	-100	1675.1
$\|9\rangle$ $\|15\rangle$ $\|7\rangle$ $\|9\rangle$	-100	1675.1	$\|3\rangle$ $\|8\rangle$ $\|5\rangle$ $\|9\rangle$	50	-639.1	$\|9\rangle$ $\|8\rangle$ $\|5\rangle$ $\|3\rangle$	-100	-1917.4
$\|3\rangle$ $\|1\rangle$ $\|13\rangle$ $\|9\rangle$	25	-717.6	$\|3\rangle$ $\|10\rangle$ $\|1\rangle$ $\|9\rangle$	25	1657.3	$\|3\rangle$ $\|14\rangle$ $\|10\rangle$ $\|9\rangle$	25	-717.6
$\|3\rangle$ $\|1\rangle$ $\|10\rangle$ $\|9\rangle$	125	1243.0	$\|3\rangle$ $\|14\rangle$ $\|13\rangle$ $\|9\rangle$	-75	3729.0	$\|3\rangle$ $\|14\rangle$ $\|1\rangle$ $\|3\rangle$	-100	2452.9
$\|9\rangle$ $\|13\rangle$ $\|10\rangle$ $\|9\rangle$	-100	2452.9	$\|3\rangle$ $\|11\rangle$ $\|2\rangle$ $\|9\rangle$	50	-834.4	$\|9\rangle$ $\|11\rangle$ $\|2\rangle$ $\|3\rangle$	-100	-2503.2
$\|0\rangle$ $\|1\rangle$ $\|13\rangle$ $\|12\rangle$	25	-615.9	$\|0\rangle$ $\|10\rangle$ $\|1\rangle$ $\|12\rangle$	25	1422.3	$\|0\rangle$ $\|14\rangle$ $\|10\rangle$ $\|12\rangle$	25	-615.9
$\|0\rangle$ $\|1\rangle$ $\|10\rangle$ $\|12\rangle$	125	1066.7	$\|0\rangle$ $\|14\rangle$ $\|13\rangle$ $\|12\rangle$	-75	3200.2	$\|0\rangle$ $\|14\rangle$ $\|1\rangle$ $\|0\rangle$	-100	1847.7
$\|12\rangle$ $\|13\rangle$ $\|10\rangle$ $\|12\rangle$	-100	1847.7	$\|0\rangle$ $\|11\rangle$ $\|2\rangle$ $\|12\rangle$	50	-705.0	$\|12\rangle$ $\|11\rangle$ $\|2\rangle$ $\|0\rangle$	-100	-2114.9
**Additional resonant terms**								
$\|i\rangle$ $\|j\rangle$		C_3	$\|i\rangle$ $\|j\rangle$		C_3			
$\|0\rangle$ $\|14\rangle$		-1066.7	$\|17\rangle$ $\|7\rangle$		-375.6			
$\|3\rangle$ $\|14\rangle$		-1243.0	$\|17\rangle$ $\|8\rangle$		-756.4			
$\|9\rangle$ $\|13\rangle$		-1243.0	$\|17\rangle$ $\|15\rangle$		-1126.8			
$\|3\rangle$ $\|16\rangle$		-967.1	$\|12\rangle$ $\|1\rangle$		1422.3			
$\|9\rangle$ $\|15\rangle$		-967.1	$\|12\rangle$ $\|2\rangle$		705.0			
$\|6\rangle$ $\|16\rangle$		-1126.8	$\|12\rangle$ $\|10\rangle$		-355.6			
$\|17\rangle$ $\|4\rangle$		1502.4	$\|12\rangle$ $\|11\rangle$		-705.0			
$\|17\rangle$ $\|5\rangle$		756.4	$\|12\rangle$ $\|13\rangle$		-1066.7			

TABLE SII. **Calculated C_3 coefficients (units of MHz μm^3) and state pair energy differences $\Delta_{ij}^{i'j'}$ (units of $2\pi\times$MHz) for all relevant dipolar exchange interaction terms by taking all Zeeman sublevels into consideration for the twisted rhombic lattice.**

our discussion only on the the case of the 12-level rhombic lattice structure. To address the effects from non-resonant state-changing interaction terms, we include all the Zeeman sublevels with $|0\rangle$ to $|11\rangle$ as defined in Fig. 1, along with an additional 6 states that are in fact not part of our synthetic lattice, which we define as: $|12\rangle = |44S_{1/2}, m_J = -1/2\rangle$, $|13\rangle = |43P_{3/2}, m_J = -3/2\rangle$, $|14\rangle = |43P_{3/2}, m_J = 3/2\rangle$, $|15\rangle = |42P_{3/2}, m_J = -3/2\rangle$, $|16\rangle = |42P_{3/2}, m_J = 3/2\rangle$, $|17\rangle = |42S_{1/2}, m_J = -1/2\rangle$. With a quantization B-field of 27 G (along $\hat{z}$, with the interatomic axis along $\hat{x}$), the C_3 coefficients and $\Delta_{ij}^{i'j'}$ for all additional resonant state-conserving and non-resonant state-changing interaction terms are

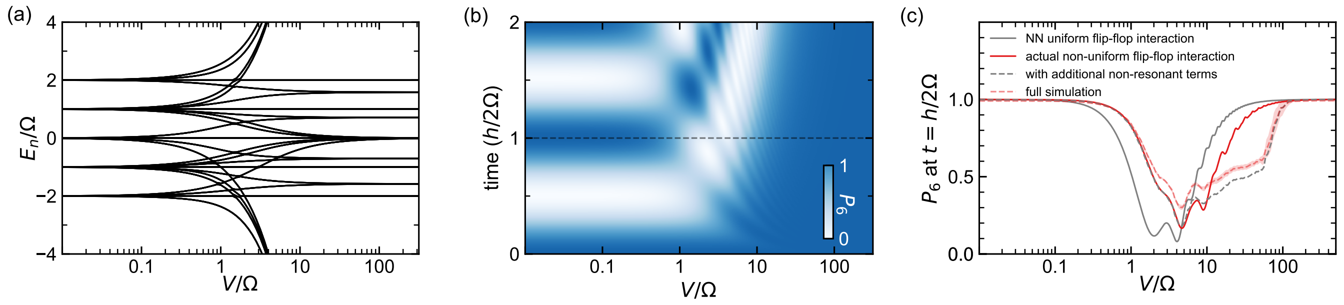

FIG. S3. **Comparison between the numerical simulation with a uniform interaction Hamiltonian and those with the physical model realized in experiment.** **(a)** Eigenenergy distribution for atom pairs in the 12-state rhombic lattice (with $\phi = \pi$ and $\theta = 0$), with an assumed uniform interaction Hamiltonian, under different interaction-to-coupling ratios V/Ω. Here n is the eigenstate index. **(b)** Time evolution of the mean population in $|6\rangle$, P_6, for different V/Ω ratios from numerical simulations with the uniform interaction Hamiltonian. The horizontal dashed line indicates the single-atom revival time. **(c)** Comparison of the P_6 populations at $t = h/2\Omega$ vs. V/Ω ratios with different numerical simulation methods. The red solid and dashed lines are identical to those shown in Fig. 3(d) in the main text. The solid black curve is for the most idealized model considered, with uniform and purely nearest-neighbor "flip-flop" interactions. The dashed black line is for the physical model with non-uniform exchange interactions, also considering non-resonant dipolar exchange terms, but ignoring non-idealities related to Rydberg state preparation infidelities, thermal variation in interparticle spacing, etc.

listed in Table SII. In these full numerical simulations, we again scale all interaction strengths $V_{iji'j'} \propto C_3^{iji'j'}/2R_{\mathrm{AB}}^3$ to the calibrated value for V_{67}.

Clarification of the simulation details on AB-caging breakdown with different Hamiltonians

We first consider a simpler but common formula of the interaction Hamiltonian $H_{\mathrm{int}} = V\sum_{\langle i,j\rangle} |i\rangle_{\mathrm{A}}\langle j| \otimes |j\rangle_{\mathrm{B}}\langle i| +$ h.c., which assumes a uniform strength for 'flip-flop' exchange process between nearest-neighboring (NN) state pairs $\langle i,j\rangle$ in Fig. 1(b). As shown in Fig. S3(a), the energy bands are much cleaner but still exhibit mixing in moderate interaction strength ($V \sim \Omega$), leading to a crossover from AB caging to interaction-induced delocalization. The larger separation of eigenstate energies at very large V/Ω leads to the interaction-inhibited transport of bound pairs for the initialized state $|6\rangle|6\rangle$. Figure S3(b) shows the time evolution of the population in $|6\rangle$. Clearly we see three different regions: (1) AB caging dynamics (oscillation between different CLSs) for small V, (2) a breakdown of AB-caging due to band mixing, and (3) bound pairs with population frozen at the initial $|6\rangle$. Finally, Fig. S3(c) shows the cut of V/Ω dependence of the P_6 population at a fixed time $h/2\Omega$, where the solid black line is for the newly considered ideal nearest-neighbor interactions. In looking at Fig. S3(c), we note that the regular wiggles or kink-like features in the P_6 line appearing as a function of V/Ω [also appearing in Fig. 3(d)] seem to stem purely from the fixed-time interference of the different two-body eigenstates onto which the initialized state $|6\rangle|6\rangle$ projects.

Next, we consider the interaction Hamiltonian $H_{\mathrm{int}} = \sum_{(i,j)} V_{ij}|i\rangle_{\mathrm{A}}\langle j| \otimes |j\rangle_{\mathrm{B}}\langle i| +$ h.c. with all possible resonant 'flip-flop' exchanges in our actual experimental realization in Fig. 1. It is employed for the numerics referred as 'ideal numerical simulations' in the main text. There are two differences from that with the above uniform NN interaction Hamiltonian: (1) the non-uniform interaction strength for each state pair based on their unique C_3 coefficients and (2) the relevant state pairs (i,j) now include ones that are 'long range', e.g., $(i = 3, j = 11)$, as listed in Table SI, due to the details of the mapping between physical internal states and the synthetic lattice sites. While the variations associated with the physical, non-uniform interactions makes the energy band mixing more complicated, we still robustly find three different regimes that indicate the crossover from AB caging, to interaction-induced delocalization, to frozen bound pairs, as shown in Figs. 3(b,c) in the main text. The specific details (onset V/Ω value and width of the breakdown region) differ from the uniform scenario, due to the details of the specific state pair interaction strengths, but the general features are quite similar.

Because we explore regimes of large interaction strengths V, and because the energy separations between the considered state pairs of our system and other configurations (including internal states that are not part of the synthetic lattice) are relatively small in our applied bias magnetic field, we have further considered the influence of non-resonant state-changing dipolar exchange processes (see Table SII), i.e., with the 'full' interaction Hamiltonian (S1). This helps to quantify the difference between our experiment measurements and the 'ideal numerical simulation' that ignores these relevant but non-ideal processes. As discussed in the above section, these terms can significantly

impact the dynamics in the large interaction ($V \gtrsim 5\Omega$) regime due to the relatively small quantization B-field in our experiment. In general, these processes can be suppressed or mitigated in several ways, either by constraining the exploration to smaller values of V or by increasing the Zeeman splitting of internal states by applying a larger quantization field (and, in one-dimensional arrays, certain dipolar terms can be suppressed by the relative orientation of the quantization field and the interatomic axis). Figure S3(c) compares the simulation results with this 'full' interaction Hamiltonian and the 'ideal simulation'. Consistent with our experimental observation, such non-resonant terms well capture the suppression of the P_6 at time $h/2\Omega$ in the region $5 \lesssim V/\Omega \lesssim 100$.

Finally, in the main text we additionally take into account the initial pair state preparation infidelity and the experimental parameter uncertainties. We use the same Monte-Carlo sample method detailed in Ref. [1] to account the impact from single atom dynamics (i.e., cases in which only one of the atoms is excited to the Rydberg state), the parameter uncertainties, and shot-to-shot interaction variations due to the finite temperature and trap release. We refer to this as 'full numerical simulation' in the main text. The simulation result is shown in Fig. 3(d) in the main text and also in Fig. S3(c) for comparison.

AB caging in the rhombic bipyramid

Aside from viewing it as a two-unit cell (with periodic boundaries) realization of the diamond lattice, the energy structure and AB caging of the rhombic bipyramid lattice (Fig. 4 in the main text) can be analyzed by viewing it as a bi-partite lattice with a chiral symmetry. Sublattice A is made of states $|1\rangle$ and $|6\rangle$, and sublattice B is made of states $|2\rangle$, $|3\rangle$, $|4\rangle$, and $|5\rangle$. Since sublattice B has two states more than sublattice A, there must be two zero modes. To note, the degeneracy at zero energy is unrelated to Aharonov-Bohm caging, appearing for all values of the flux on the faces of the bipyramid structure.

Since there is a chiral symmetry in the model, the entire spectrum will be particle-hole symmetric; if there is a state with energy E, one obtains another state with energy $-E$ by adding minus signs (π phase shifts) to the wavefunction amplitudes on the A sublattice sites. Now what is left is to understand the two-fold degeneracy at nonzero energies. This part is where the Aharonov–Bohm (AB) caging becomes relevant. First of all, nonzero energy states should have some wavefunction on both the A and B sublattices. Because of the π-flux through the faces of the bipyramid, there is a state which has nonzero wavefunction amplitude on state $|1\rangle$ but zero wavefunction on state $|6\rangle$. Because of the up-down symmetry of the structure (up to a gauge transformation that changes the location of the phase-inverted hopping terms), there is a state with the same energy whose wavefunction on state $|6\rangle$ is nonzero but is zero on state $|1\rangle$. (These two states are linearly independent because one has no wave function on state $|6\rangle$ but the other has nonzero wavefunction on state $|6\rangle$.) Thus the degeneracy at nonzero energy is the result of the Aharonov-Bohm caging combined with the Z_2 symmetry (up-down symmetry) of the structure; similar degeneracy in AB caging configurations due to spatial symmetries has been previously discussed in Refs. [2–4].

Derivation of emergent magnetism from interacting flat bands

The non-interacting single particle Hamiltonian for the rhombic bipyramid structure shown in Fig. 4(b) of the main text reads

$$H_{\rm sp} = \frac{\Omega}{2} \sum\nolimits_{j=2}^{5} (|1\rangle\langle j| + e^{i\phi_{6j}} |j\rangle\langle 6|) + \text{h.c.} \tag{S2}$$

with $\phi_{62} = \phi_{64} = \pi$ and $\phi_{63} = \phi_{65} = 0$. As shown in Fig. 4(c) of the main text, this Hamiltonian has three sets of degenerate pairs of CLSs: (i) The upper $+\Omega$ energy band with wavefunctions $|\lambda_u^-\rangle = \frac{1}{\sqrt{8}}(-2|1\rangle - |2\rangle - |3\rangle - |4\rangle - |5\rangle)$ and $|\lambda_u^+\rangle = \frac{1}{\sqrt{8}}(-2|6\rangle + |2\rangle - |3\rangle + |4\rangle - |5\rangle)$. (ii) The middle zero energy band with $\left|\lambda_0^-\right\rangle = \frac{1}{10}(-|2\rangle - 7|3\rangle + |4\rangle + 7|5\rangle)$ and $\left|\lambda_0^+\right\rangle = \frac{1}{10}(-7|2\rangle + |3\rangle + 7|4\rangle - |5\rangle)$. (iii) The lower $-\Omega$ energy band with $\left|\lambda_l^-\right\rangle = \frac{1}{\sqrt{8}}(2|1\rangle - |2\rangle - |3\rangle - |4\rangle - |5\rangle)$ and $\left|\lambda_l^+\right\rangle = \frac{1}{\sqrt{8}}(2|6\rangle + |2\rangle - |3\rangle + |4\rangle - |5\rangle)$. Relevant for the description of emergent ground state magnetism, one can encode an effective spin-1/2 into the two degenerate states in the lowest flat band as [see Figs. 4(c,d)]

$$\begin{aligned} |\downarrow\rangle &= \left|\lambda_l^-\right\rangle, \\ |\uparrow\rangle &= \left|\lambda_l^+\right\rangle. \end{aligned} \tag{S3}$$

To note, the states $|\downarrow\rangle$ and $|\uparrow\rangle$ are also referred to in the main text as $|\Psi_\downarrow\rangle$ and $|\Psi_\uparrow\rangle$, respectively.

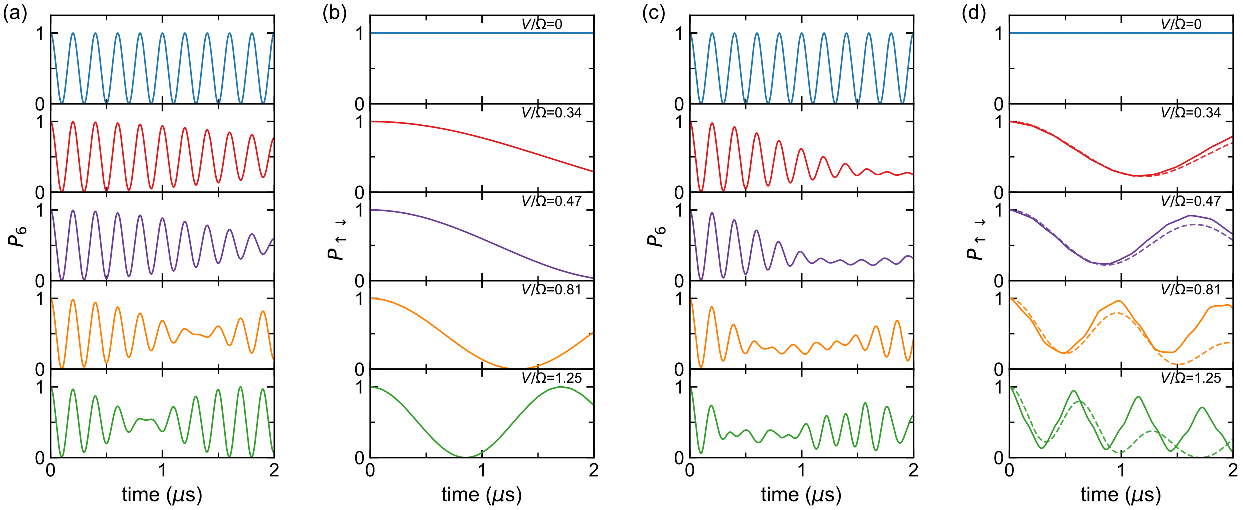

FIG. S4. **Comparison of the dynamics based on Hamiltonians (S2) and (S5): (a,b) with a uniform interaction $3V/16$ which leads to Hamiltonian (S7) excluding the $\sigma_x\sigma_z$ term; (c,d) with the real interaction used in the experiment, i.e., Eq. (S7) with all interaction terms.** **(a)** "Ideal model" time evolution of the population in $|6\rangle$ under different interactions, with the initial state $|6\rangle_\mathrm{A}|6\rangle_\mathrm{B}$. **(b)** "Ideal model" time evolution of the population in $|\uparrow\downarrow\rangle$, with the initial state $|\uparrow\downarrow\rangle = |\lambda_l^+\rangle_\mathrm{A}|\lambda_l^-\rangle_\mathrm{B}$; see (S3). With no fast oscillations (because the population is largely restricted to a single band), the dynamics is fully determined by $P_{\uparrow\downarrow}(t) = \cos^2(2J_\mathrm{XX}t/\hbar) = \cos^2(2J_\mathrm{eff,id}t/\hbar)$ with the effective spin-exchange interaction strength $J_\mathrm{eff,id} = J_\mathrm{XX} = 3V/64$. **(c)** "Physical model" time evolution of the population in $|6\rangle$ under different interactions, with the initial state $|6\rangle_\mathrm{A}|6\rangle_\mathrm{B}$. With additional effective $\sigma_x\sigma_z$ terms, the full dynamics are modified from the simple beating formula of (S6). The $\sigma_x\sigma_z$ terms only change the envelope of the beating; the fast dynamics still have an oscillation frequency of $2\Omega/\hbar$, as seen by comparing (a) and (c). **(d)** "Physical model" time evolution, with initial conditions and plotted observable similar to those in panel (b). The added $\sigma_x\sigma_z$ terms increase the overall rate of intra-band pseudospin dynamics. By diagonalizing the effective spin Hamiltonian (S7), we find that the dynamics should follow the formula $P_{\uparrow\downarrow}(t) = \frac{3}{8} + \frac{1}{4}\cos(2(J_\mathrm{XZ} - 2J_\mathrm{XX})t/\hbar) + \frac{1}{4}\cos(2(J_\mathrm{XZ} + 2J_\mathrm{XX})t/\hbar) + \frac{1}{8}\cos(4J_\mathrm{XZ}t/\hbar)$ [$\sim \cos^2(2J_\mathrm{eff}t/\hbar)$ in short time limit, with $J_\mathrm{eff} = \sqrt{J_\mathrm{XX}^2 + J_\mathrm{XZ}^2/2}$], shown as dashed lines. Short-time fits to Eq. (S6) should reveal a low frequency component $\omega \approx J_\mathrm{eff}$. For large V, the analytical results (dashed lines) deviate from the numerical simulation with the original Hamiltonian (solid lines), due to the onset of band mixing as V approaches the band gap, Ω (see Fig. 4(e) of the main text). For all simulations here, we use $\Omega/h = 2.5$ MHz and $V/\Omega = \{0, 0.34, 0.47, 0.81, 1.25\}$ from top to bottom panels.

To obtain the effective magnetic Hamiltonian for an atom pair (labelled as A and B), we expand the interaction Hamiltonian

$$H_\mathrm{int} = \sum\nolimits_{j=2}^{5} \left(V_{6j}|6\rangle_\mathrm{A}\langle j| \otimes |j\rangle_\mathrm{B}\langle 6| + V_{1j}|1\rangle_\mathrm{A}\langle j| \otimes |j\rangle_\mathrm{B}\langle 1|\right) + \mathrm{h.c.} \tag{S4}$$

under the spin basis $\{|\downarrow\downarrow\rangle, |\downarrow\uparrow\rangle, |\uparrow\downarrow\rangle, |\uparrow\uparrow\rangle\}$. For uniform interaction strength, i.e., $V_{6j} = V_{1j} = V$ for $j \in [2,5]$, in the weak interaction limit, it takes the following form

$$H_\mathrm{int} = \frac{V}{4}(\sigma_\mathrm{A}^z\sigma_\mathrm{B}^z + \mathbb{1}_\mathrm{A}\mathbb{1}_\mathrm{B}) + \frac{V}{4}(\sigma_\mathrm{A}^x\sigma_\mathrm{B}^x + \sigma_\mathrm{A}^y\sigma_\mathrm{B}^y). \tag{S5}$$

In this ideal case, the effective spin exchange rate is $2J_\mathrm{eff,id} = 2J_\mathrm{XX}$, with $J_\mathrm{eff,id} = J_\mathrm{XX} = J_\mathrm{ZZ} = V/4$ being the common prefactor of the XX, YY, and ZZ terms. To note, the constant $\mathbb{1}_\mathrm{A}\mathbb{1}_\mathrm{B}$ term does not affect the physics of this system, and is omitted in the main-text description of the effective spin model. With the initial population all in state $|6\rangle$ (the initial condition in our experiment), the dynamics of this ideal model follows

$$P_6(t) = \frac{1}{2} + \frac{1}{2}\cos\left(\frac{2J_\mathrm{eff,id}}{\hbar}t\right)\cos\left(\frac{2\Omega}{\hbar}t\right), \tag{S6}$$

which is actually a beating formula between two oscillation frequencies:

(i) one is the inter-band oscillation from the single particle Hamiltonian H_sp, since the initial state $|6\rangle$ is a superposition of $|\lambda_l^+\rangle$ and $|\lambda_u^+\rangle$, i.e., $|6\rangle = \frac{1}{\sqrt{2}}(|\lambda_l^+\rangle - |\lambda_u^+\rangle)$. These two eigenstates have an energy difference of 2Ω, which naturally makes the population in $|6\rangle$ oscillate with a frequency of $2\Omega/\hbar$.

(ii) another is the interaction-induced effective spin exchange with the rate $2J_{\text{eff,id}}/\hbar$.

For our real, physical system shown in Fig. 4(a) of the main text, the interaction strengths of the various Rydberg state pairs are not uniform (in the synthetic dimension). We scale all of them to the strongest one, i.e., letting $V_{62} = V$, leading to $\{V_{62}, V_{63}, V_{64}, V_{65}\} = \{V, -V/4, V/2, -V/2\}$ and $\{V_{12}, V_{13}, V_{14}, V_{15}\} = \{-V/4, V, -V/2, V/2\}$ from the calculated C_3 coefficients. This experimental interaction Hamiltonian can be expanded in spin basis as

$$H_{\text{int}} = \frac{3V}{64}(\sigma^z_{\text{A}}\sigma^z_{\text{B}} + \mathbb{1}_{\text{A}}\mathbb{1}_{\text{B}}) + \frac{3V}{64}(\sigma^x_{\text{A}}\sigma^x_{\text{B}} + \sigma^y_{\text{A}}\sigma^y_{\text{B}}) + \frac{9V}{64}(\sigma^x_{\text{A}}\sigma^z_{\text{B}} + \sigma^z_{\text{A}}\sigma^x_{\text{B}}). \tag{S7}$$

The effective spin exchange rate is $2J_{\text{XX}}$ with $J_{\text{XX}} = J_{\text{ZZ}} = 3V/64$. However, the interaction Hamiltonian also includes an additional XZ term, which induces correlated spin-flip oscillations ($|\uparrow\uparrow\rangle \leftrightarrow -|\uparrow\downarrow\rangle$, $|\uparrow\uparrow\rangle \leftrightarrow -|\downarrow\uparrow\rangle$, $|\downarrow\downarrow\rangle \leftrightarrow |\uparrow\downarrow\rangle$, and $|\downarrow\downarrow\rangle \leftrightarrow |\downarrow\uparrow\rangle$) that consequently affect the P_6 dynamics. By comparing Hamiltonians (S7) and (S5), the beating dynamics should be modified [with the appropriate form given in the caption of Fig. S4(d)], with multiple low-frequency contributions owing to the added presence of the J_{XZ} terms. In our experiment, we simply fit the P_6 dynamics to the form $P_6(t) = a + b\cos\left(\frac{2\omega}{\hbar}t\right)\cos\left(\frac{2\Omega}{\hbar}t\right)$ inspired by Eq. (S6). These fits yield a trend of ω values that is in fair agreement with the analytical expression for the effective interaction scale that accounts for the influence of the J_{XZ} terms, given by $J_{\text{eff}} = \sqrt{J^2_{\text{XX}} + J^2_{\text{XZ}}/2} \propto V$. This approximate form is shown as the solid line in Fig. 4(g) of the main text. We find that the experimentally observed rates ω are also in good agreement with the expected response based on analogous fits of the numerically simulated dynamics, which is shown as the dashed-dotted line in Fig. 4(g). To note, the linear scaling of J_{eff} with V is expected to break down due to band mixing as V approaches Ω, which is reflected in the prediction based on numerical simulations.

[1] Tao Chen, Chenxi Huang, Ivan Velkovsky, Kaden R. A. Hazzard, Jacob P. Covey, and Bryce Gadway, "Strongly interacting rydberg atoms in synthetic dimensions with a magnetic flux," Nature Communications **15**, 2675 (2024).

[2] Julien Vidal, Benoît Douçot, Rémy Mosseri, and Patrick Butaud, "Interaction induced delocalization for two particles in a periodic potential," Phys. Rev. Lett. **85**, 3906–3909 (2000).

[3] B. Douçot, M. V. Feigel'man, and L. B. Ioffe, "Topological order in the insulating josephson junction array," Phys. Rev. Lett. **90**, 107003 (2003).

[4] Benoit Douçot, Lev B. Ioffe, and Julien Vidal, "Discrete non-abelian gauge theories in josephson-junction arrays and quantum computation," Phys. Rev. B **69**, 214501 (2004).
